# Investigating the impact of alumina nanoparticles in coconut oil distillate biodiesel to lessen emissions in direct injection diesel engine

**DOI:** 10.1038/s41598-024-63862-7

**Published:** 2024-06-09

**Authors:** K. Rajesh, Chidambaranathan Bibin, Gopinath Soundararajan, R. Ashok Kumar, S. Arunkumar, Yuvarajan Devarajan, Nandagopal Kaliappan

**Affiliations:** 1 Engineering Division, Unitive Technologies Pvt. Ltd, Chennai, Tamilnadu India; 2 Department of Mechanical Engineering, R.M.K. College of Engineering and Technology, Chennai, Tamilnadu India; 3 Department of Mechatronics Engineering, Rajalakshmi Engineering College, Chennai, Tamilnadu India; 4 Department of Mechanical Engineering, RMD Engineering College, Chennai, Tamilnadu India; 5 Department of Mechanical Engineering, Thiagarajar College of Engineering, Madurai, Tamilnadu India; 6 Department of Mechanical Engineering, Saveetha School of Engineering, SIMATS, Saveetha University, Chennai, Tamilnadu India; 7 Department of Mechanical Engineering, Haramaya Institute of Technology, Haramaya University, Dire Dawa, Ethiopia

**Keywords:** Waste-to-Energy, Sustainable Practices, Methyl ester, Emissions, Renewable energy, Engineering, Mechanical engineering

## Abstract

Petroleum fuels are commonly used for automobiles. However, the continuous depletion and exhaust gas emission causes serious problems. So, there is a need for an alternative eco-friendly fuel. Biodiesel is a type of fuel manufactured through a process called transesterification, which involves converting vegetable oils into a usable form. The process parameters of the transesterification process were optimized using the Taguchi method to achieve maximum biodiesel yield. However, the main problem of biodiesel is its high cost which could be reduced by using low-cost feedstock. To address this challenge, biodiesel (BCFAD) is derived from coconut fatty acid distillate (CFAD), a by-product obtained from refining coconut oil. This work uses BCFAD and BCFAD with Alumina nanoparticles as fuels. Alumina nanoparticles in the mass fraction of 25 ppm, 50 ppm, and 100 ppm are dispersed in BCFAD. The investigation results reveal an increase of 6.5% in brake thermal efficiency for BCFAD with 100 ppm nanoparticles when compared to BCFAD. There is a reduction of 29.29% of hydrocarbon and 34% of Carbon monoxide emissions with BCFAD100 in comparison with diesel. However, there is a marginal increase in NOx emission with the increase in nanoparticles. The heat release rate and cylinder pressure of BCFAD100 are comparable to diesel fuel. It was concluded that the utilization of BCFAD with a nanoparticle dispersion of 100 ppm is suitable for direct use as fuel in diesel engines.

## Introduction

The world majorly depends on fossil fuels for its energy requirements. However, using fossil fuels leads to continuous depletion, cost increases, and pollutes the environment^1^. Vegetable oils are widely used to produce biodiesel, which is considered the best alternative to diesel fuel. However, the production is not economical due to the current manufacturing and inventory network^2^. To overcome the drawback, biodiesel from waste and residual by-products is considered the best alternative^3^. The fatty acid distillates (FAD) obtained during fatty acid stripping and deodorization of vegetable oil are a low-value by-product^4^. The FAD is used as raw material in soap, animal food, and chemical industries^5^. It can also be used to produce low-cost biodiesel^6^. Around the world, several thousand metric tons of FAD from different feedstocks are produced annually^7^. Hence, FAD can be used to produce.

Coconut oil is used for many applications, and refining it produces FAD^8^. Coconut oil fatty acid distillate (CFAD) is a suitable feedstock to produce biodiesel at a low cost^9^. Engine performance and component life are greatly affected by using vegetable oils or fatty acid distillates as fuel^10^. To overcome the problems of obtaining eco-friendly, economical, and easily available fuel, it is necessary to alter the physiochemical properties of vegetable oils or FAD^11^. One of the methods to achieve change in physiochemical properties is the transesterification process^12^. The methyl ester (BCFAD) derived from CFAD used in diesel-fuelled engines reduces emissions, but the performance reduces due to the lower heating value^13^. The nanoparticle additives reduce engine emissions and fuel consumption. Hence, BCFAD can be used along with fuel additives to improve the engine performance^14^.

An extensive study was conducted to examine the performance, combustion characteristics, and emissions of diesel engines fuelled with biodiesel blended with alumina nanoparticles at different molar concentrations^15^. Notable improvements were observed in specific fuel consumption (SFC), brake thermal efficiency (BTE), and heat release rate were revealed as the nanoparticle concentration increased^16^. Likewise, a slight enhancement in brake thermal efficiency and emissions was observed in a direct injection (DI) diesel engine when employing alumina nanoparticles and carbon nanotubes as additives alongside biodiesel and its blend. Importantly, superior performance with biodiesel blends, as compared to both neat diesel and biodiesel alone, was observed for these additives^17^.

Furthermore, the impact of cerium oxide nanoparticles added to biodiesel revealing significant improvements in thermal efficiency, flash and fire points, and reduced emissions of hydrocarbons (HC) and nitrous oxide (NO)^18^. In another study, the effects of zinc oxide (ZnO) nanoparticles added to diesel fuel in varying concentrations in single-cylinder direct-injection diesel engines were investigated and concluded that ZnO nanoparticles increased thermal efficiency but resulted in a notable increase in NOx emission compared to neat diesel^19^.

The collective literature suggests that the addition of nanoparticles into biodiesel fuel may have positive effects on combustion. However, recent examining the experimental performance and emissions of BCFAD blended with nanoparticles. Therefore, the impact of alumina nanoparticles in BCFAD on the performance, combustion, and emissions of a diesel engine is being explored in the present experimental work.

Alumina nanoparticles were added into BCFAD at concentrations of 25 ppm, 50 ppm, and 100 ppm. The investigation involved combustion characteristics such as cylinder pressure and heat release rate, along with engine performance parameters including brake thermal efficiency (BTE) and specific fuel consumption (SFC). Additionally, the assessment of CO, NOx, and HC emissions will be conducted.

## Materials and methods

### Biodiesel preparation

The process of transesterification is the most suitable method for producing biodiesel from FAD of vegetable oil with methanol^20^. This process is employed to reduce the viscosity of the FAD by reacting it with methanol in the presence of a KOH catalyst^21^. The coconut oil FAD was obtained from a local retailer in Chennai. Catalyst (KOH in pellet form) and methanol of analytical reagent grade are used. The titration procedure evaluates the amount of free residual acidity in CFAD. The required amount of CFAD is heated in a container using a temperature control heater maintained at 62 °C. Methanol and KOH catalysts are mixed in a conical flask and transferred to the heated CFAD in the container. Using a mechanical stirrer at a controlled speed of 600 rpm, the CFAD and Methanol with catalyst were mixed to a homogeneous composition. The process is continued as the fatty acids are converted into methyl esters. Then it is transferred to the separation flask, the heavier crude glycerine settles at the bottom, and the methyl ester (BCFAD) at the top. The glycerine is removed and the methyl ester is rinsed until all the soap content is removed. The process parameters of the transesterification process were optimized using the Taguchi method to achieve maximum biodiesel yield. Four parameters and three levels were selected for the study. Based on the L9 orthogonal array, nine experiments were carried out with variations in the parameters and levels. The yield of all the experiments was tabulated and the optimum parameter values were obtained based on the signal to noise ratio. Based on the optimum parameters experiments were conducted and experimental results were compared with predicted results.

### Preparation of fuel blend with nanoparticles

The BCFAD is mixed with aluminium oxide nanoparticles in 25 ppm, 50 ppm, and 100 ppm mass fractions. 25 mg of Al_2_O_3_ nanoparticle was added to 1 L of BCFAD to make the dosing level 25 ppm (BCFAD25). Similarly, for 50 ppm and 100 ppm, the dosing level is increased to 50 mg/l and 100 mg/l, respectively. The mixture is shaken well and agitated for about 30 min in an ultrasonic agitator to uniformly suspend nanoparticles. Before being used in the engine, the mixture is thoroughly shaken to ensure even distribution, as nanoparticles tend to settle in the solution.

### Properties with nanoparticle samples

The properties of the samples were assessed using an appropriate method, and the results are presented in Table 1. The flash and fire points of biodiesel were determined using an open cup flash and fire point apparatus^22^. The density of the blend was determined using a hydrometer maintained at a temperature of 39 °C. The kinematic viscosity of the biodiesel blend at a specific temperature was measured using a redwood viscometer. The calorific value of the fuel blend was determined according to the ASTM D240 standard using a bomb calorimeter.

**Table 1:** Properties of diesel and biodiesel blend.

Properties	Diesel	BCFAD	BCFAD25	BCFAD50	BCFAD100	Test method
Kinematic viscosity @ 40 °C in Cst	2.84	4.63	4.58	4.47	4.30	ASTM D445
Flash Point (°C)	50	54	55	57	59	ASTM D93
Calorific Value (kJ/kg)	42.7	33.8	33.9	34.1	34.7	ASTM D240
Density (kg/m^3^)	833	870	874	877	879	ASTM D127

## Experimental setup and measurements

**Figure 1:**
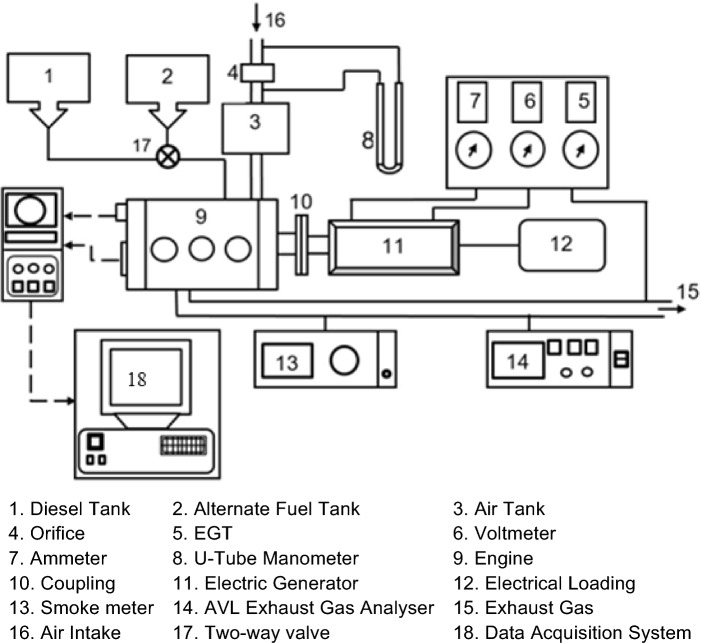
A schematic of the diesel engine used for the study.

Experiments were conducted using a Kirloskar AV1 water-cooled four-stroke diesel engine, with a rated power of 5.20 kW and operating at a constant speed of 1500 rpm. Figure 1 illustrates the experimental setup, where the engine is connected to a swinging field electrical generator equipped with a loading system. The specifications of the direct injection (DI) diesel engine utilized in the experiment are outlined in Table 2.

**Table 2:** Engine specification.

Type	Kirloskar AV 1, Water cooled, Four Stroke
Number of cylinders	Single
Bore	87.5 mm
Compression ratio	17.5:1
Maximum power	5.20 kW
Speed	1500 rpm
Dynamometer	Electrical
Injection pressure	200 bar

Emissions of HC, CO, and NOx were measured using the AVL-444 gas analyser, while smoke opacity was monitored using the AVL-437 Hatridge smoke meter. K-type thermocouples were utilized to measure exhaust gas temperature, and in-cylinder pressure at each crank angle (CA) was recorded using a piezoelectric pressure sensor coupled with a charge amplifier transducer within the 0–100 bar range. Data acquisition from the engine, including engine speed, fuel flow, and emission characteristics, was facilitated by the data acquisition system. The engine performance is analysed from the collected data and the combustion characteristics were documented for biodiesel with various nanoparticle concentrations.

### Uncertainty analysis

A particular amount of uncertainty always exists in any type of measurement of an experiment, regardless of the instrument type used. The uncertainty resulting from any measurement may arise because of fixed or random errors. Uncertainties and errors in the measurements can arise due to various factors like instrument selection, calibration, environmental condition, testing procedures, observation method, interpretation etc. The analysis was performed to validate the reliability of the measured value of the instrument. In this study, the percentage relative uncertainty of engine load, speed, exhaust temperature, fuel consumption, HC, CO, NOx and smoke opacity were calculated using the linearized approximation method of uncertainty. The overall uncertainty of the experimental investigation was found after calculating the individual uncertainty of the measured parameters using Eq. (1).

$${\text{Overall}}\,{\text{uncertainty}} = \sqrt {\sum \left( {Uncertainty\,of\,each\,parameter} \right)^{2} }$$

1 $$\begin{aligned} {\text{Overall}}\,{\text{uncertainty }} & = {\text{square}}\,{\text{root}}\,{\text{of}}\,[({\text{engine}}\,{\text{load}})^{2} + ({\text{engine}}\,{\text{speed}})^{2} + {\text{(time)}}^{2} \\ & \quad + ({\text{exhaust}}\,{\text{temperature}})^{2} + ({\text{Engine}}\,{\text{power}})^{2} + ({\text{fuel}}\,{\text{flow}})^{2} + ({\text{BTE}})^{2} \\ & \quad + ({\text{BSEC}})^{2} + ({\text{HC}})^{2} + ({\text{CO}})^{2} + ({\text{NOx}})^{2} + ({\text{Smoke}}\,{\text{opacity}})^{2} \\ & = {\text{Square}}\,{\text{root}}\,{\text{of}}\,[(0.2)^{2} + (1.0)^{2} + (0.2)^{2} + (0.1)^{2} + (1.0)^{2} + (1.0)^{2} + (1.0)^{2} \\ & \quad + (1.5)^{2} + (0.2)^{2} + (0.2)^{2} + (0.2)^{2} + (1.0)^{2} \\ & = \pm 2.73\% \\ \end{aligned}$$

## Results and discussion

A four-stroke single-cylinder direct injection water-cooled diesel engine was used for the experiment. Testing was performed on the engine using pure diesel, BCFAD, and BCFAD with nanoparticles dispersed in proportions of 25, 50, and 100 ppm. The performance, emission, and combustion characteristics were analysed and compared to diesel.

### Performance characteristics

#### Specific fuel consumption

**Figure 2:**
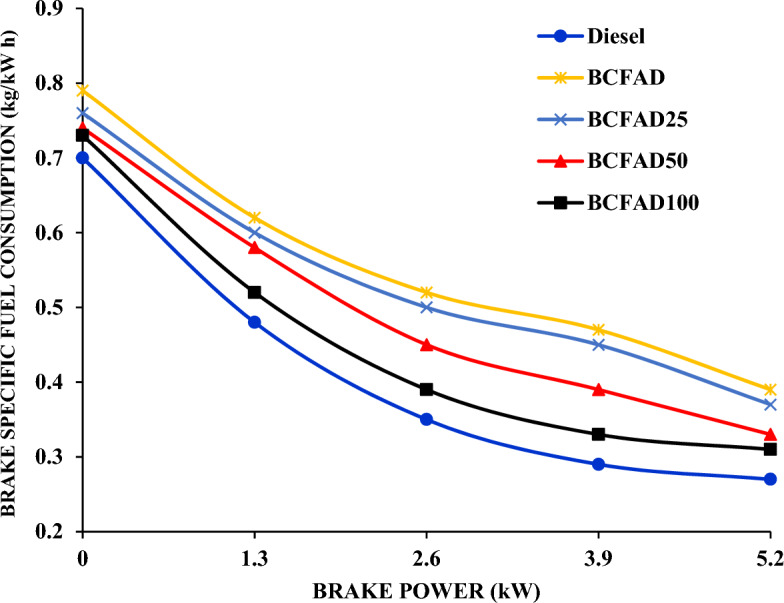
Specific fuel consumption against brake power.

The SFC of biodiesel with Al_2_O_3_ nanoparticles blends of different dosing levels, BCFAD, and diesel with load is shown in Fig. 2. The fuel consumption of BCFAD is higher than BCFAD25, BCFAD50, BCFAD100, and diesel for all loads. BCFAD exhibits a lower calorific value in comparison to diesel fuel, resulting in higher fuel consumption to generate equivalent power output. The SFC decreases as the dosing level of Al_2_O_3_ nanoparticles increases. The decrease in SFC may be due to the presence of nanoparticles in the blend as it possesses a better catalytic effect, enhanced area–volume ratio, and less fuel consumption^23^. The addition of nanoparticles in BCFAD resulted in a considerable reduction of 7.66% in SFC compared to neat biodiesel at 75% of the load.

#### Brake thermal efficiency

**Figure 3:**
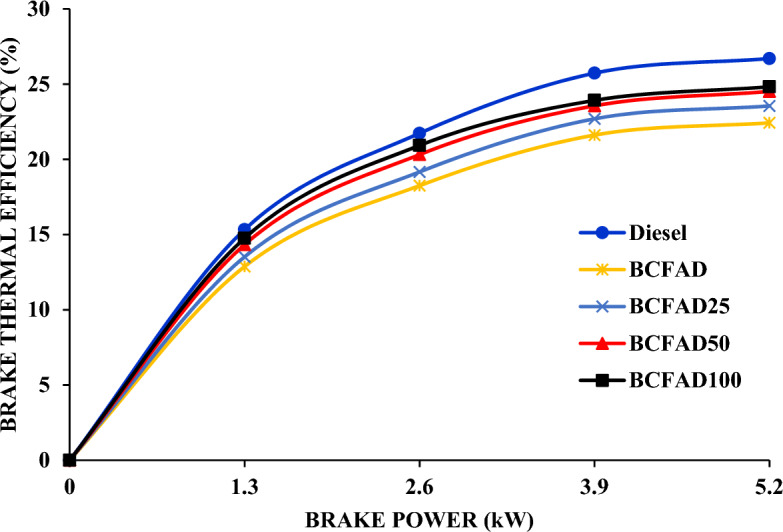
Brake thermal efficiency against brake power.

Figure 3 illustrates the impact of load variation on brake thermal efficiency. It depicts the ratio of brake power to the energy released in a combustion process. The engine test result shows that the BTE is improved by adding Al_2_O_3_ nanoparticles when tested with different biodiesel blends. Complete combustion is achieved in the biodiesel blend due to the presence of Al_2_O_3_ nanoparticles, contrasting with sole biodiesel. The Al_2_O_3_ nanoparticles serve as an oxygen buffer, reducing ignition delay, accelerating the burning rate, and consequently enhancing brake thermal efficiency^24^. It is inferred that the BTE shows a marginal increase with the concentration level of the nanoparticles^25^. BCFAD with 100 ppm nanoparticles results in a 6.5% increase in BTE compared to neat biodiesel.

### Emission characteristics

#### Carbon monoxide

**Figure 4:**
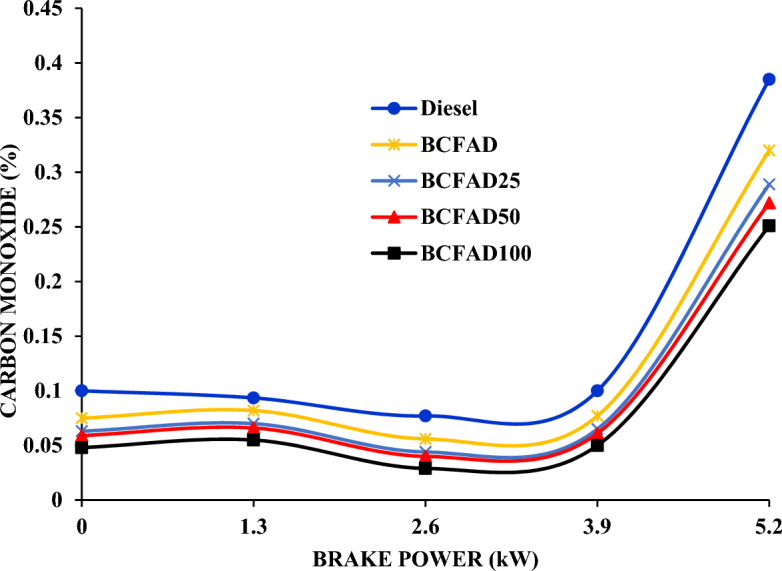
Carbon monoxide emission against brake power.

The variation of carbon monoxide (CO) levels in diesel, biodiesel, and their blends concerning load is illustrated in Fig. 4. CO may be due to air–fuel ratio, injection pressure, and engine speed. As discussed earlier, oxygen content in biodiesel leads to better combustion than diesel resulting in reduced CO emission. The nanoparticles act as a catalyst, leading to better combustion as the concentration increases^26^. The nanoparticle concentration shortens the ignition delay, leading to better air–fuel mixing and enhanced combustion^27^. Thus, there was a notable decrease in CO emission with the metal oxide blended BCFAD. The CO emission does not vary much in diesel, biodiesel, and biodiesel blends. At 100% load, the CO increases for all the fuels due to more fuel supply to the engine to maintain the power output^28^. A large reduction is observed at 100% load condition for diesel and BCFAD100.

#### Hydrocarbon

**Figure 5:**
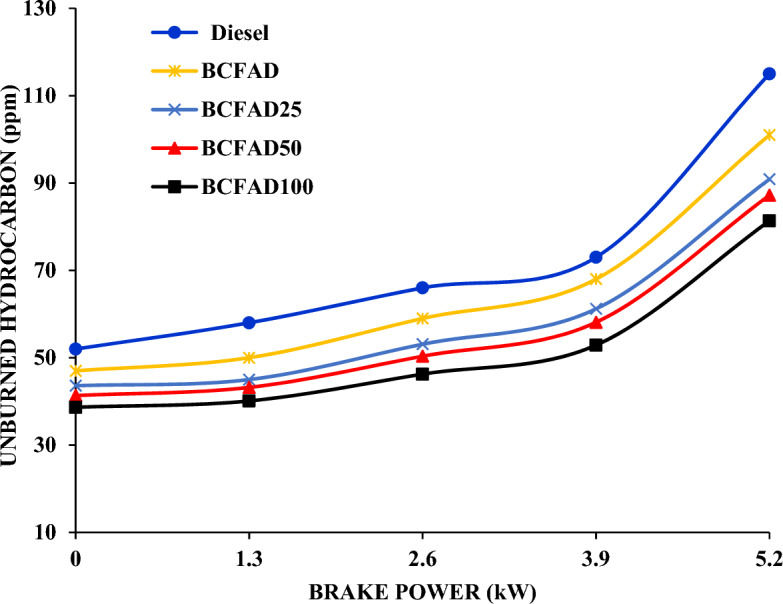
Unburnt Hydrocarbon against brake power.

Figure 5 compares hydrocarbon (HC) emissions and diesel, biodiesel, and biodiesel blends. The HC emission decreases with the increasing concentration of aluminium oxide nanoparticles. The HC increases with the engine load for diesel, BCFAD, and BCFAD blends. The nanoparticle increases the level of oxygen content BCFAD blends and it is the main reason for the reduction in HC and enhanced combustion^29^. HC emission was 115 ppm, 101 ppm, 90.9 ppm, 87.23 ppm, and 81.36 ppm at full load condition.

#### Oxides of nitrogen

**Figure 6:**
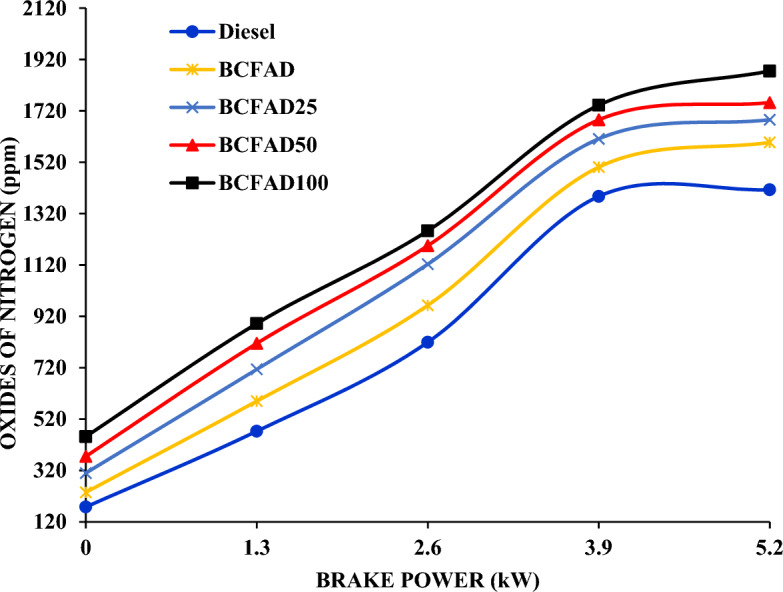
Oxides of Nitrogen against brake power.

Figure 6 displays the nitrogen oxide emissions relative to load for various fuel blends. It is noticed that NOx emission increases for biodiesel because of more oxygen content in the biodiesel than diesel. The inclusion of nanoparticles in BCFAD further increases the combustion rate, resulting in more NOx formation^30^. This was due to higher operating temperature, the most favourable condition for inert nitrogen to react with oxygen to form NOx^31^. Among all the fuels tested, BCFAD25 has lower emissions compared to diesel.

### Combustion characteristics

#### Cylinder pressure

**Figure 7:**
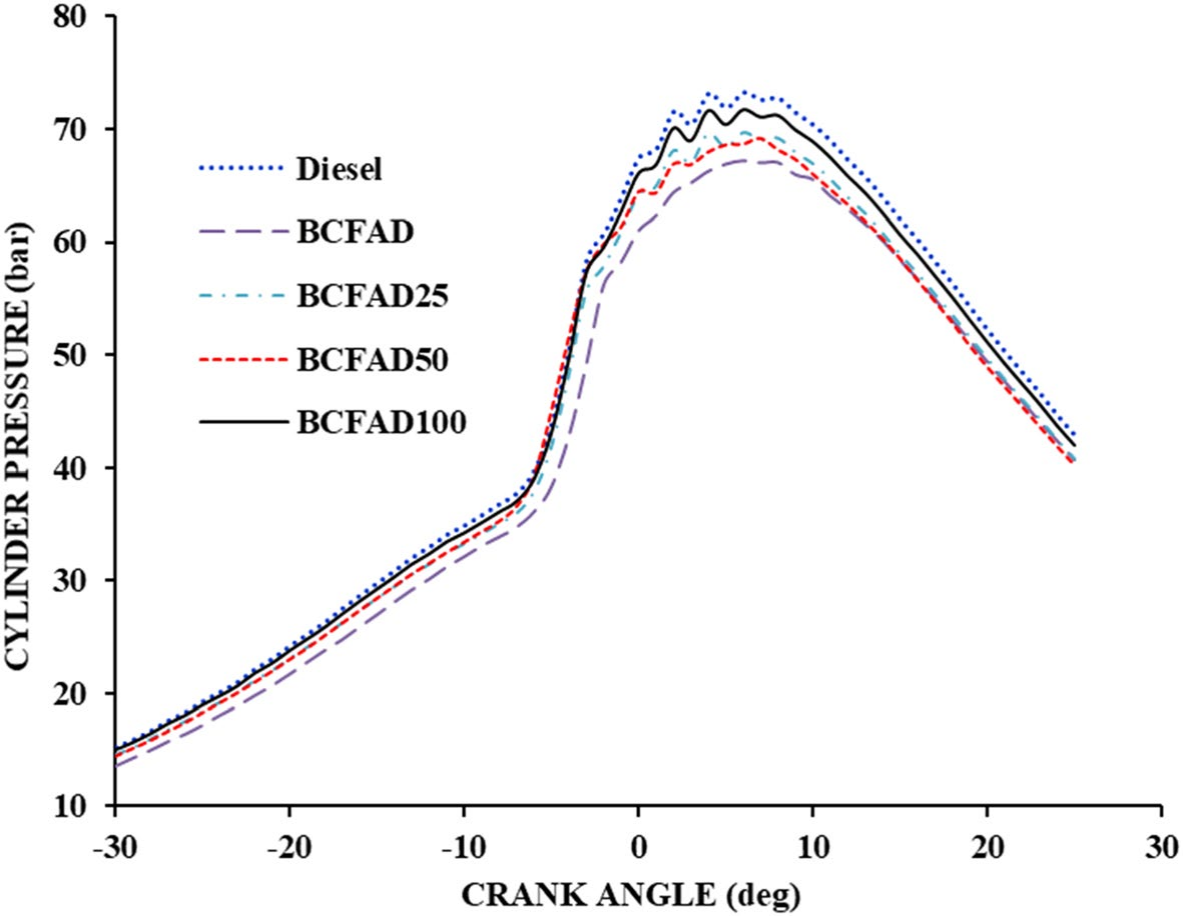
Cylinder pressure against crank angle.

The combustion characteristics of BCFAD fuel and its blends are expressed by cylinder peak pressure with crank angle, as shown in Fig. 7. The ability of the fuel to mix with air results in an improved combustion rate, which leads to increased cylinder gas pressure. The cylinder wall temperature and exhaust gas temperature decrease with the reduction in engine load because of injection timing and ignition delay^32^. The nanoparticles added to biodiesel improve combustion by reducing the ignition delay^33^. It is observed that the cylinder gas pressure is higher for BCFAD100 than BCFAD but lower for neat diesel.

#### Heat release rate

**Figure 8:**
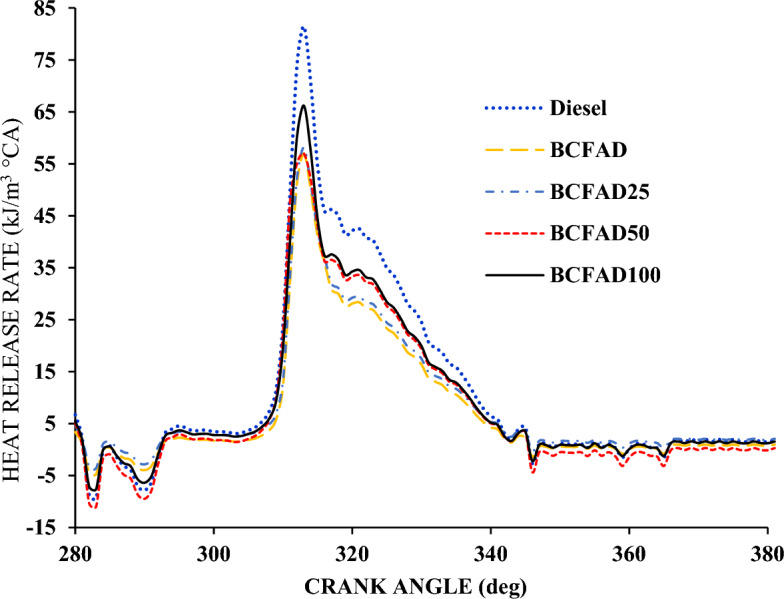
Heat release rate against crank angle.

Figure 8 presents a comparison of the heat release rate (HRR) at full load condition with respect to crank angle. It is observed that the HRR is negative at the beginning of combustion for the fuel due to the vaporization of the fuel and the heat lost to the coolant from the engine through cylinder walls. The diesel fuel has a higher HRR than BCFAD and its blends since the heating value of BCFAD is lower than diesel. A significant increase in the HRR was found with the improvement in the dosage of nanoparticles^34^. The nanoparticles shorten the ignition delay period and accelerate the combustion, increasing HRR for BCFAD^35^. It is noted that the HRR of BCFAD100 is closer to neat diesel fuel.

#### Combustion duration

**Figure 9:**
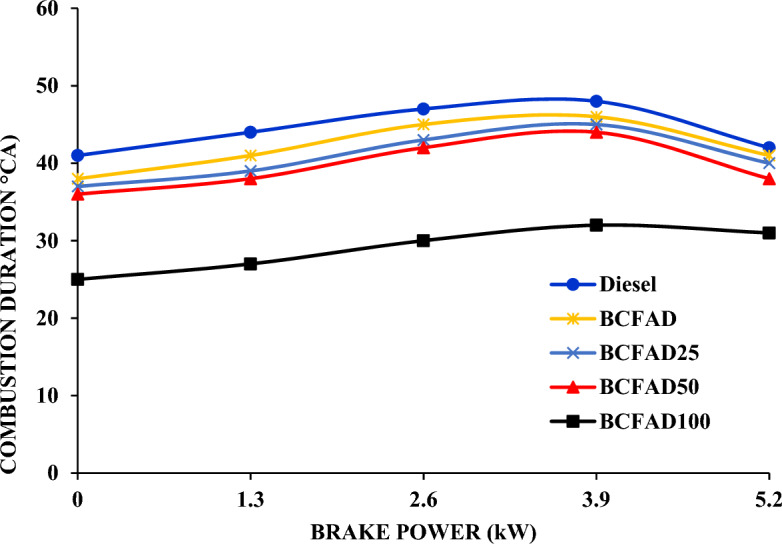
Combustion duration against brake power.

Figure 9 shows the comparison of combustion duration of the BCFAD and its diesel blends with diesel at various loads. The combustion duration for BCFAD blends is significantly higher than for diesel. Superior viscosity and low volatility of BCFAD oil improve the distribution of the size of droplet of its combustion chamber fuel spray^36,37^. Bigger size droplets of comparatively inferior volatility of fuel take a long time for fuel atomization and associated combustion^38^. Therefore, the introduction of even small amounts of BCFAD oil in the test fuel will induce a fairly excessive rise in the duration of the combustion.

## Cost analysis

The assessment of a new alternative fuel's viability often hinges on life cycle cost estimates, which take into account factors like the fuel's energy demands, input from feedstocks, and environmental impacts. Conducting such an analysis can yield valuable insights into the energy consumption, raw material requirements, and waste generation at each stage of the biodiesel production process.

Life cycle cost analysis essentially involves a structured model that factors in equipment and process costs, as well as the operational lifespan of a manufacturing plant's production facility. India stands out as one of the world's major consumers of petroleum products, with over 80% of its demand being met through imports from other countries.

In terms of feedstock, studies have highlighted that the life cycle cost of CFAD biodiesel is notably lower compared to Jatropha biodiesel, especially when considering identical feedstock costs. Further investigation revealed that the expenses related to biodiesel raw materials, followed by operational expenditures, contribute significantly to savings, accounting for more than 75% of the overall life cycle cost.

## Conclusion

Based on the results obtained from the present investigation, the DI diesel is significantly influenced by the addition of nanoparticles in the characteristics of biodiesel. The following conclusions were made from the performance, emission, and combustion characteristics of different fuel blends.

- Nanoparticles blended with BCFAD show notable improvements in specific fuel consumption (SFC) compared to pure BCFAD, yet they still fall short of diesel's performance. The decrease in SFC is attributed to the catalytic influence and the surface area-to-volume ratio of the nanoparticles.
- Increasing the nanoparticle dosage in BCFAD leads to a proportional increase in brake thermal efficiency (BTE). For instance, BCFAD100 exhibits a 6.5% rise in BTE compared to regular BCFAD.
- Both BCFAD and nanoparticle-blended fuel exhibit significantly reduced hydrocarbon (HC) and carbon monoxide (CO) emissions compared to diesel.
- However, nitrogen oxide (NOx) emissions tend to rise with biodiesel and its blends due to quicker combustion, resulting in higher operational temperatures and increased oxygen levels.
- Cylinder pressure during combustion is lower for biodiesel and its blends than for diesel, primarily because their combustion initiates earlier, reducing peak pressure near top dead centre (TDC).
- Diesel fuel outperforms BCFAD in terms of heat release rate (HRR) due to its higher energy content. The addition of nanoparticles increases the HRR compared to plain BCFAD.

It is clear from the results that the aluminium oxide nanoparticles (Al_2_O_3_) when blended with biodiesel, improve the fuel properties. The fuel can be used in diesel engines with a 100 ppm dosage of nanoparticles for better performance and emission characteristics. The increase in NOx may be reduced by emulsification or modified injection strategies like multiple injections.

## Data Availability

The datasets used and/or analysed during the current study available from the corresponding author on reasonable request.

## Abbreviations

FAD: Fatty acid distillate
CFAD: Coconut fatty acid distillate
BCFAD: Coconut fatty acid distillate biodiesel
HC: Unburnt hydrocarbons
CO: Carbon monoxide
NOx: Oxides of nitrogen
SFC: Specific fuel consumption
BTE: Brake thermal efficiency
DI: Direct injection
BCFAD25: BCFAD + 25 ppm of Al_2_O_3_
BCFAD50: BCFAD + 50 ppm of Al_2_O_3_
BCFAD100: BCFAD + 100 ppm of Al_2_O_3_
HRR: Heat release rate
